# Epicutaneous Immunotherapy (EPIT) Blocks the Allergic Esophago-Gastro-Enteropathy Induced by Sustained Oral Exposure to Peanuts in Sensitized Mice

**DOI:** 10.1371/journal.pone.0031967

**Published:** 2012-02-21

**Authors:** Lucie Mondoulet, Vincent Dioszeghy, Thibaut Larcher, Mélanie Ligouis, Véronique Dhelft, Emilie Puteaux, Yan Cherel, Franck Letourneur, Christophe Dupont, Pierre-Henri Benhamou

**Affiliations:** 1 DBV Technologies, Pépinière Santé Paris Cochin, Paris, France; 2 INRA, UMR703, National Veterinary School, Nantes, France; 3 Institut Cochin, Université Paris Descartes, INSERM-U567, Paris, France; 4 Université Paris Descartes - Hôpital Necker-Enfants Malades, Paris, France; University Hospital Freiburg, Germany

## Abstract

**Background:**

Food allergy may affect the gastrointestinal tract and eosinophilia is often associated with allergic gastrointestinal disorders. Allergy to peanuts is a life-threatening condition and effective and safe treatments still need to be developed. The present study aimed to evaluate the effects of sustained oral exposure to peanuts on the esophageal and jejunal mucosa in sensitized mice. We also evaluated the effects of desensitization with epicutaneous immunotherapy (EPIT) on these processes.

**Methods:**

Mice were sensitized by gavages with whole peanut protein extract (PPE) given with cholera toxin. Sensitized mice were subsequently exposed to peanuts *via* a specific regimen and were then analysed for eosinophilia in the esophagus and gut. We also assessed mRNA expression in the esophagus, antibody levels, and peripheral T-cell response. The effects of EPIT were tested when intercalated with sensitization and sustained oral peanut exposure.

**Results:**

Sustained oral exposure to peanuts in sensitized mice led to severe esophageal eosinophilia and intestinal villus sub-atrophia, *i.e.* significantly increased influx of eosinophils into the esophageal mucosa (136 eosinophils/mm^2^) and reduced villus/crypt ratios (1.6±0.15). In the sera, specific IgE levels significantly increased as did secretion of Th2 cytokines by peanut-reactivated splenocytes. EPIT of sensitized mice significantly reduced Th2 immunological response (IgE response and splenocyte secretion of Th2 cytokines) as well as esophageal eosinophilia (50 eosinophils/mm^2^, p<0.05), mRNA expression of Th2 cytokines in tissue - eotaxin (p<0.05), IL-5 (p<0.05), and IL-13 (p<0.05) -, GATA-3 (p<0.05), and intestinal villus sub-atrophia (2.3±0.15). EPIT also increased specific IgG2a (p<0.05) and mRNA expression of Foxp3 (p<0.05) in the esophageal mucosa.

**Conclusions:**

Gastro-intestinal lesions induced by sustained oral exposure in sensitized mice are efficaciously treated by allergen specific EPIT.

## Introduction

Digestive lesions such as eosinophilic gastrointestinal disorders (EGID) and food-induced enteropathy, with more or less pronounced villus atrophy (VA), are usually related to food-allergen exposure [1]–[3]. Treatment consists of elimination of the offending food(s) [2]–[4], using more or less elemental formula —often poorly tolerated— and local or systemic steroids, [5]. The last is an effective treatment but has side effects including reduction of height gain in children and triggering of esophageal candidiasis [6]. Discontinuation of any of the current treatment regimens can result in relapse [7], indicating a need for alternative treatments.

Epicutaneous immunotherapy (EPIT) has gained increasing evidence for safety and efficacy in the treatment of allergy in animals [8], [9] and humans [10], [11]. Until now, its potential role in treating eosinophilic esophagitis (EoE) and allergic enteropathy has not been investigated. The first clinical trials of oral specific immunotherapy had promising results for food allergy but revealed EoE as a potential side-effect [12], [13].

In mice, EPIT depressed the eosinophilic infiltration of the lung after nasal challenge through a Treg-dependant mechanism of down-regulating the Th2 biased immune response [8], [9]. However, the effect of EPIT on the esophageal and intestinal mucosa after sustained oral exposure to allergens in sensitized mice has never been described.

The present study thus aimed to evaluate the effects of sustained oral exposure to peanuts on the esophageal and jejunal mucosa in sensitized mice that were desensitized with EPIT. For this purpose, we developed a murine model of sustained oral exposure to peanuts in sensitized mice, resulting in esophageal eosinophilia and intestinal villus sub-atrophia. We believe that this model is efficaciously mimicking homologous human conditions, and can be proposed to test innovative interventions in the field of specific immunotherapy. Mice were submitted to an elimination diet of the offending food followed by sustained oral exposure to allergens. We took advantage of this peculiar configuration to analyze the mucosal reaction when the sustained food challenge is preceded by EPIT.

## Materials and Methods

### Animals

Three-week-old female BALB/c mice (Charles Rivers, Lyon, France) were purchased and housed under standard animal husbandry conditions. All experiments were performed according to the European Community rules on animal care, with permission 92–305 from the French Veterinary Services and with a positive evaluation from the Ethical Committee of Paris Descartes University (Paris, France; P2.LM.130.10). Mice were acclimated for 1 week before immunization.

### Food sensitization and sustained oral peanut exposure (figure 1)

#### Set up of the model (figure 1A)

**Figure 1:**
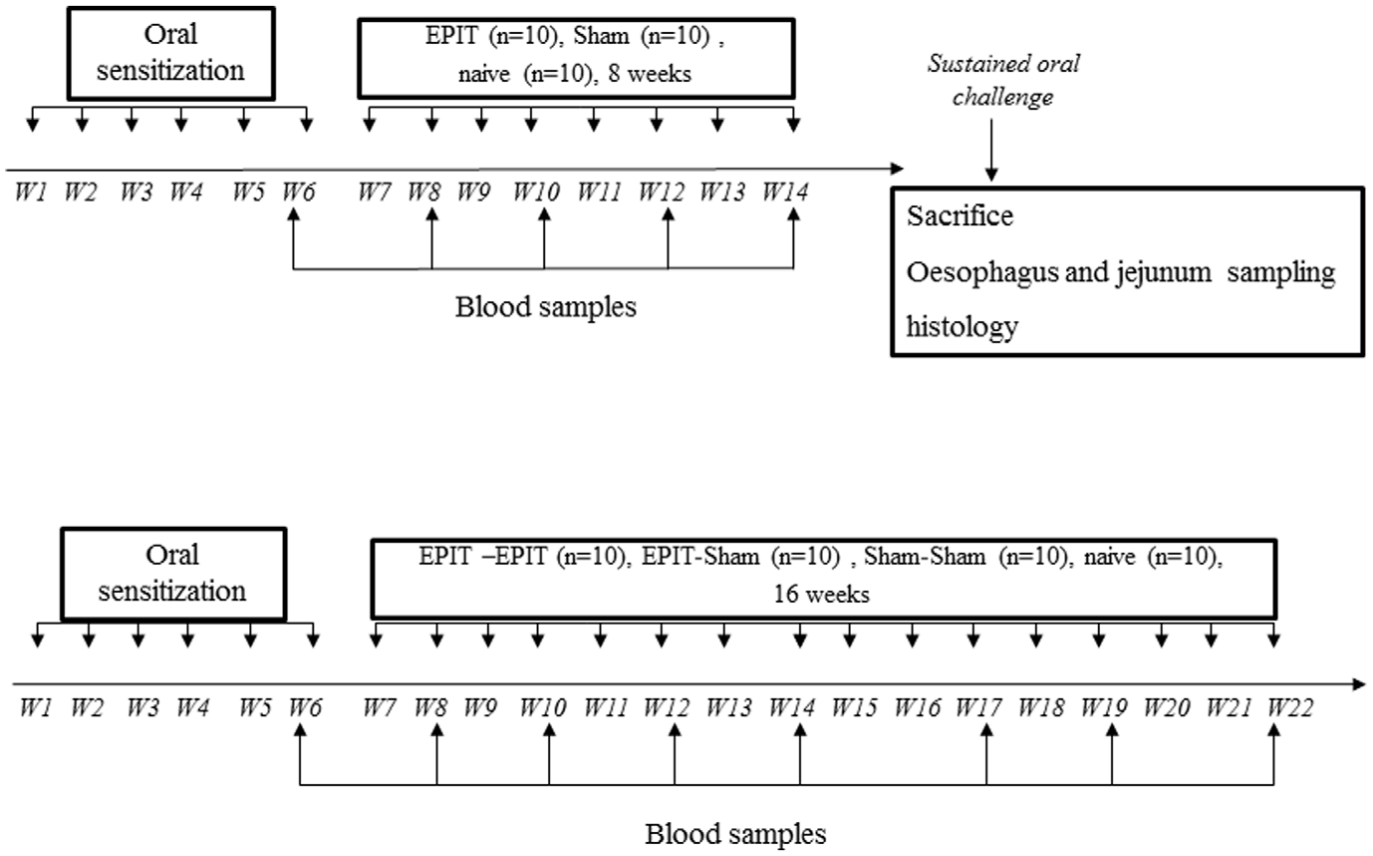
Study design for induction of eosinophilic esophagatis and enteropathy and for the effect of EPIT on the induction of digestive lesions. (A) Fourty mice were sensitized to peanut proteins in the first phase. Then a resting period with no treatment and no peanut administration was applied. After that, a peanut regimen for 10 days was given to sensitized and naïve mice (n = 40). Mice were then sacrificed to analyze esophagus and jejunum samples by histology and RT-qPCR. (B) Twenty mice were sensitized to peanut proteins in the first phase. Epicutaneous immunotherapy was conducted for 8 weeks in 1à sensitized mice (EPIT) and 10 other sensitized mice received a Sham treatment (Sham). After a sustained oral challenge, mice were sacrificed to analyze esophagus and jejunum samples by histology and RT-qPCR. Blood samples were taken every 2 weeks to measure specific immunoglobulins (IgE, IgG1, IgG2a).

To evaluate the lesions triggered by a sustained oral exposure to peanuts, 40 mice were first sensitized to peanut proteins (PPE) by means of 6 intra-gastric gavages (D1, D7, D13, D19, D25, D32), as previously described [9], with 1 mg of PPE mixed with 10 µg of Cholera Toxin (CT – Servibio, USA). Mice were then maintained for 8 weeks on a peanut-free regimen without any treatment. After that, peanuts were reintroduced into their regimen (sustained oral exposure to peanuts). This consisted of exclusive feeding with peanut seeds instead of standard mouse food for 4 consecutive days. As all mice (including naïve mice) exhibited diarrhea may be due to the highly fat enriched feeding, animals then received peanut seeds mixed into standard food for the 6 following days, and daily intra-gastric administration of a solution containing 10 mg of peanut protein for the last 3 days of this second phase. At different time points during peanut regimen (before, D2, D4, D7, and D10), 8 sensitized mice were sacrificed after deep anesthesia with an intra-peritoneal injection of 30 mg/kg sodium pentobarbital and samples were taken to examine the histology of the esophagus and jejunum, the mRNA expression of cytokines in the esophagus mucosa, and the cytokines secreted by reactivated splenocytes. Blood samples were also collected from retro-orbital plexus under anesthesia with isoflurane (Isoflurane Belamont, Nicholas Piramal India) at the end of the sensitization period (D44) and just before the beginning of the sustained oral exposure to peanuts (D89). Forty naive mice (5×8 mice) serving as controls were also subjected to the same procedures.

#### Intercalation of epicutaneous immunotherapy (EPIT) (figure 1B)

As a second investigation, the effects of sustained oral exposure to peanuts using the above protocol were evaluated following an 8-week period of desensitization by EPIT. For this study, 10 mice were sensitized to peanut proteins and treated with EPIT and compared with sensitized and non-treated animals (Sham). The animals then underwent sustained oral exposure to peanuts. Ten naive mice serving as controls received the same procedures. The day after the last challenge, mice were anesthetized and sacrificed and sample studies were performed as described above.

### Analysis of esophageal eosinophilia and jejunum villus atrophy

Esophagus and jejunum were collected and fixed in 4% neutral-buffered formalin, embedded in paraffin wax, sliced transversally into 5-µm thick sections, and fixed onto positive-charge slides. Sections were then stained using a routine hematoxylin-eosin-safranine staining method (HES).

Three sections of esophagus and 6 sections of jejunum were analyzed in a double-blind manner. All lesions were reported by a skilled ECVP-certified pathologist. Semi-quantitative evaluations of the mucosal thickness of the esophagus reflecting acanthosis and chorionic inflammation in the esophagus and jejunum were also performed. Thickness scores were as follows: 0, no more than 3 layers of acanthocytes; 1, from 4 layers of acanthocytes; and 2, more than 6 layers of acanthocytes with diffuse hyperkeratosis. Inflammation was scored was as follows: 0, less than 2 eosinophils per high-powered field; 1, diffuse infiltration by scattered eosinophils; 2, presence of foci of more than 20 eosinophils; and 3, presence of foci of more than 100 eosinophils.

Image analysis was performed on esophagus and jejunum sections using a digital camera (Nikon DXM 1200, Champigny, France) combined with image-analysis software (Nikon Imaging Software) used only for photo acquisition and data storing. Six high-powered fields were randomly selected around the esophageal lumen and quantification of thickness was performed from the lumen to the basement membrane of each esophagus. Eosinophils, characterized by their pink-red granulation and their horseshoe-shaped bilobed nucleus, were then counted by the pathologist, and results were expressed as number of eosinophils per mm^2^. Intra-observer agreement was tested by reproducing this measure 3 times on the same sample and the coefficient of reproducibility was determined to be 93.7%. In addition, 6 intermediate-powered fields were randomly selected around the jejuna lumen, and the ratio of villous height to crypt depth was evaluated. Intraobserver agreement was tested by triplicate measures of eachsample. Reproducibility was 89.9%.

### Epicutaneous immunotherapy (EPIT)

EPIT was performed using the EDS Viaskin® (DBV Technologies, Paris France) [14] and the treatment protocol which has been previously described [9]. Briefly, mice were anaesthetized intraperitoneally with 100 mg/kg body weight of ketamine (Imalgen1000®, Merial, Lyon, France) and 10 mg/kg body weight xylazine (Rompun®, Bayer, Puteaux, France), and were then shaved with an electric clipper and depilatory cream without corticoid (Veet®, Massy, France). Twenty-four hours later, after total recovery of the skin, mice were anaesthetized and EDS with 100 µg of PPE, provided by Greer Laboratories (Lenoir, USA), were applied once a week for 48 h to the back of mice from which hair had been removed. This technique for the preparation of the skin does not modify the barrier properties of the skin. This was demonstrated in a preliminary experiment where transepithelial water loss (TEWL) after skin preparation was measured and compared with the values obtained in hairless mice (6.45±1.22 *vs.* 6.63±1.49 g/h/m^2^ repectively, ns). For sham immunotherapy, the EDS was left empty, and no treatment was administered to the naive group.

### Reverse-transcription quantitative PCR (RT-qPCR)

Total RNA from esophageal sections was extracted using the RNeasy Mini Kit (Qiagen, Courtaboeuf, France) in accordance with the manufacturer's instructions. The concentration of RNA was determined and complementary DNA (cDNA) was synthetized by reverse transcription reaction (SuperScript II RNase H reverse transcription reagents, Invitrogen, Cergy-Pontoise, France) containing 500 ng of RNA from the experimental sample. Quantitative PCR analyses in real time were performed with the LightCycler® 480 Real-Time PCR System using SYBR-green fluorescence (Roche Diagnostic, Mannheim, Germany) for quantification. The thermal-cycling conditions were: 95°C for 5 min and then 45 cycles at 95°C (5 s), 55°C (5 s.), and 72°C (10 s.). This was followed by the standard denaturation curve. The murine primer sequences are shown in Table 1. These were designed with the OLIGO6 software package using the nucleotide sequences from the GenBank database. Each PCR reaction contained LightCycler® 480 SYBR Green I Master Mix (Roche Diagnostic), the gene specific primers, and the cDNA derived from the experimental RNA sample. The threshold for positivity was determined based on the negative controls. Results were presented as mRNA expression in the naive, EPIT, and Sham animals. Target gene expression was calculated relative to the expression of *β-actin* and *SDHA* in each experimental sample, using the ΔCq method and the results were depicted as arbitrary units. In a preliminary study, we validated that *β-actin* and *SDHA* are the 2 most stable housekeeping genes (HKG) in our model and could be used together to normalize mRNA expression data. Each set of quantitative PCR reactions were also ran with negative controls without RNA and without RT.

**Table 1:** Primer sequences used in quantitative real time-PCR assays.

Primers	Sequences	Predicted fragment length
β-actine	Sense GTGGCATCCATGAAACTACATAntisense GGCATAGAGGTCTTTACGG	73 bp
SDHA	Sense CTTGAATGAGGCTGACTGTGAntisense ATCACATAAGCTGGTCCTGT	87 bp
TGF-β	Sense TGACGTCACTGGGAGTTGTACGGAntisense GGTTCATGTCATGGATGGTGC	170 bp
IFN-γ	Sense TCAAGTGGCATAGATGTGGAAGAAAntisense TGGCTCTGCAGGATTTTCATG	93 bp
IL-4	Sense CATGGGAAAACTCCATGCTTAntisense ATGAATCCAGGCATCGAAAA	87 bp
IL-5	Sense GCTGGCCTCAAACTGGTAATGTAAntisense GGCAATGGTGCATGTCTGTAACCTC	100 bp
IL-13	Sense AGACCAGACTCCCCTGTGCA Antisense TGGGTCCTGTAGATGGCATTG	125 bp
IL-10	Sense CCAGAGCCACATGCTCCTAGA Antisense AGCTGGTCCTTTGTTTGAAAGAA	78 bp
CCL-11 (eotaxin)	Sense AAACAACCTCCTCTCTTGACACTAAAntisense GCGACTGGTGCTGATATTCC	114 bp
Foxp3	Sense CCCGGAGAGGCAGAGGACACTCAAT Antisense AGGCTCAGGTTGTGGCGGATG	114 bp

### Measurement of blood-specific IgE, IgG1, and IgG2a

Blood was collected after sensitization (week 6), and every 2 weeks during immunotherapy (*i.e.* weeks 8, 10, 12, and 14) and plasma samples were prepared in tubes containing EDTA.

Specific antibodies were quantified using a quantitative ELISA developed in-house according to the 2001 FDA guidelines as previously described [8]. Briefly, plasma samples were incubated in microtiter plates previously coated with PPE. The presence of specific IgE (sIgE), IgG1 (sIgG1), and IgG2a (sIgG2a) was detected by the addition of an anti-mouse IgE, IgG1 or IgG2a antibody labeled with alkaline phosphatase (Serotec, Oxford, England). The reagent, pNPP (Sigma, France) was used as an enzyme substrate and the optical density was measured at 405 nm. Specific IgE, IgG1, and IgG2a were quantified by comparison with concentration-response curves obtained with a total IgE, IgG1 or IgG2a assay performed under identical conditions using a solid phase coated with an anti-mouse IgE, IgG1 or IgG2a antibody (Serotec, Oxford, England) -which is complementary to tracer- instead of peanut proteins. Mouse immunoglobulin standards were obtained from Serotec (Oxford, England).

As the high level of IgG might lead to underestimation of the sIgE level, the ELISA method has been confirmed by a reverse enzyme allergo-sorbent assay (EAST).

### Cytokine production

After 8 weeks of treatment, spleens were teased into a single-cell suspension and washed three times in RPMI-1640 (Gibco, France). Cells were counted and adjusted to a culture density of 4×10^6^ cells/ml and cell suspensions of 500 µl were placed into each well of a 24-well microtitre plate (Nunc) together with 500 µl medium or PPE (100 µg/ml). After 72 h, the supernatants were harvested and analysed for the presence of cytokines (IL-4, IL-5, IL-13, IL-10, IFN-γ) using the BioPlex cytokine Assay (BioRad, Marnes-la-Coquette, France) in accordance with the manufacturer's instructions. TGF-β was analysed using ELISA kit (R&D system, Minneapolis, USA).

### Statistical analysis

The GraphPad Prism Software 5.0 (San Diego, CA, USA) was used for statistical analysis (n = 10 mice per group). Results are expressed as mean ± standard deviation (SD). Antibody and cytokine responses were analyzed using analysis of variance (ANOVA) and Tukey's test for intergroup comparisons. For histological analyses, statistical significance comparing different sets of mice was determined by Student's *t* test.

## Results

### The model of peanut-induced allergic esophago-gastro-enteropathy

**Figure 2:**
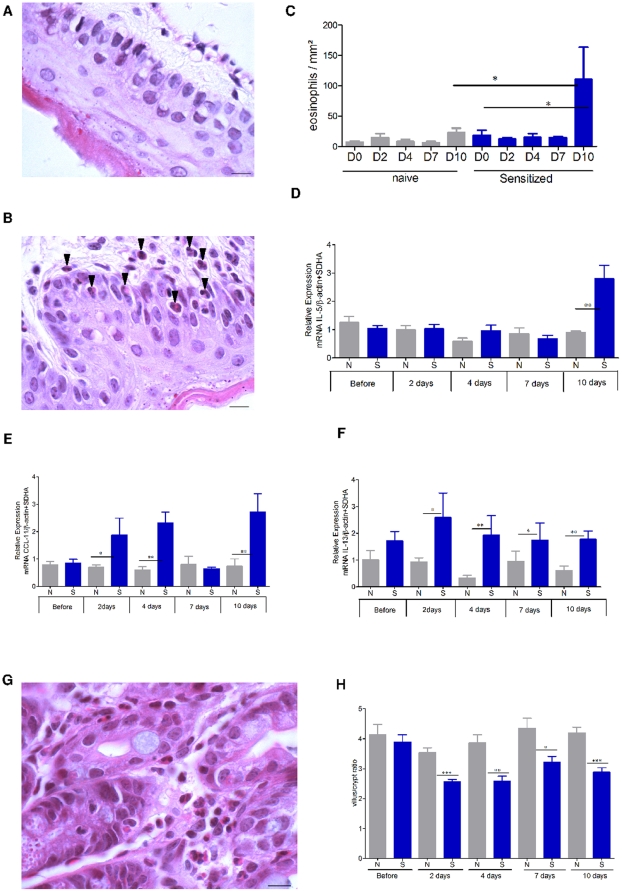
Establisment of the model of peanut-induced allergic esophagus-gastro-enteropathy by histological and RT-qPCR analyses. Microscopic analysis of eosinophils at 100× in the esophagus of naive (A) and sensitized (B) mice. Most eosinophils (arrows) are located in the lamina propia, submucosa, and epithelial layer of the sensitized group. (C) Measurement of eosinophil infiltration in the esophagus at 40×; the results are expressed as number of eosinophils per mm^2^ (means ± SD). Cytokine mRNA from esophagus segments was assayed by RT-qPCR. The relative levels of gene expression were calculated by reference to the mRNA levels of SDHA and β-actin in each sample. (D) IL-5, (E) eotaxin, (F) IL-13. Jejunum segments collected and analyzed by microscopy after HES coloration (×40); inflammatory infiltration, particularly of eosinophils is shown in (G). Measurement of the ratio of villous height to crypt depth for each group of 10 mice at 10×. Results are expressed as means ± SD (H). N: naïve mice, S: sensitized mice.* p<0.05, ** p<0.01, *** p<0.001.

The kinetics of digestive, esophageal and jejunal, lesions triggered by the sustained oral exposure to peanuts were easily monitored as indicated in the Methods section (Figure 2).

To a clinical point of view, all mice (sensitized or not) had seborrhea and diarrhea without any differences between groups. No weight loss nor significant changes in activity/nutritional status nor drop of rectal temperature were observed.

In peanut-sensitized mice, only 10 days after the sustained oral exposure to peanuts, the esophagus showed massive infiltration with inflammatory cells, particularly eosinophils, in the lamina propria around the vascular plexus or more diffusely in the most severe cases (Figures 2A and 2B). The eosinophil infiltration increased from nil at day 0 (before sustained oral exposure to peanuts) to 110.5 eosinophils/mm^2^ after 10 days; this was significantly higher than in naive mice (22.6 eosinophils/mm^2^, p<0.05) (Figure 2C). Eosinophil infiltration was accompanied by an increased esophageal expression of IL-5, IL-13, and eotaxin mRNA (Figures 2D, 2E, and 2F). The significant induction of IL-5 for sensitized mice appeared after 10 days of sustained oral exposure to peanuts (2.8 vs 0.89 for naïve, p<0.01) (Figure 2D). From 2 days of peanut regimen to the end of peanut regimen, eotaxin and IL-13 mRNA were significantly over-expressed in sensitized mice compared with naive mice.

In the jejunum, sustained oral exposure to peanuts was associated with obvious jejunal lesions (Figure 2G), with deep necrotic debris in the crypts of Lieberkuhn. Compared with naive mice, the recruitment of inflammatory cells in the lamina propria consisted mostly of eosinophils in the sensitized mice. In the most severe cases, large groups of clustered eosinophils reached into the submucosa, some of them migrating across the epithelium. From the second day to the end, the sustained oral exposure to peanuts also induced a degree of villous sub-atrophy in the sensitized mice with an overall decrease in villous height and increase in crypt depth, which significantly reduced the villus/crypt ratio compared with naive mice (Figure 2H).

**Figure 3:**
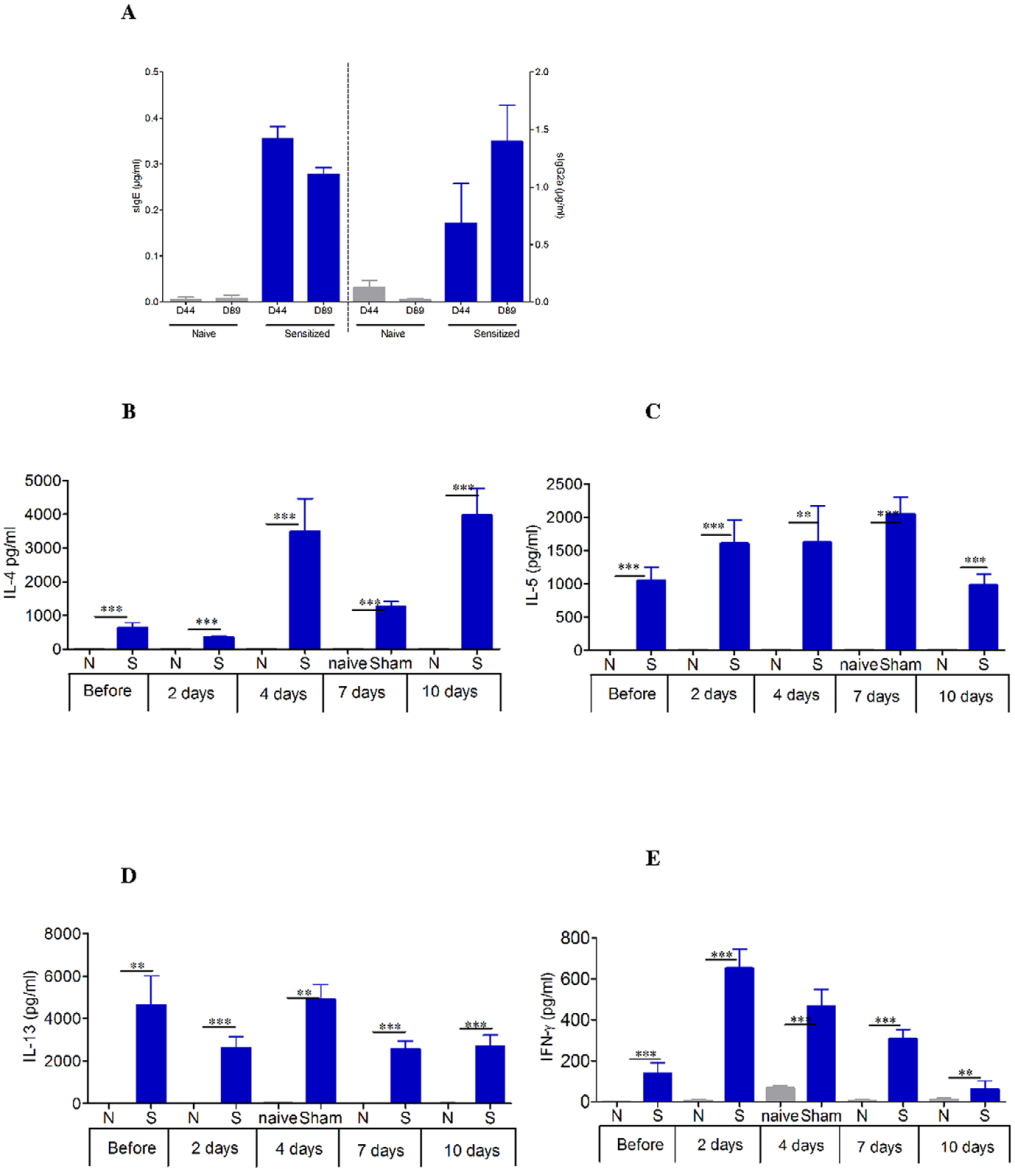
Systemic response induced in mice after oral sensitization analyzed in plasma and spleens. (A) Quantity of specific IgE and IgG2a expressed in µg.ml^−1^ for each group. Data are expressed as means ± SD for each group mice, D44 after oral sensitization, D89 after the 8-week resting period of peanut free diet. (B–E) Measurement of Th2 cytokine levels (IL-4, IL-5, IL-13) and IFN-γ secretion by splenocytes collected from each group of mice (EPIT, Sham, and naïve) immediately after sacrifice. Splenocytes were prepared and stimulated with PPE for 72 h. Cytokines were measured by ELISA. Data are presented as means ± SD for each group of mice. N: naïve mice, S: sensitized mice. ** p<0.01, *** p<0.001.

sIgE, sIgG1, and sIgG2a levels were monitored after sensitization and every 2 weeks during the 8 weeks of no treatment combined with peanut elimination (figure 3). After sensitization, sIgE increased in all sensitized mice (figure 3A), as previously observed [8], [9]. During the 8 weeks of peanut food exclusion, specific IgE as well as specific IgG2a did not change from D44 to D89 (Figure 3A). In response to PPE stimulation, splenocytes in culture showed markedly increased levels of the Th2 cytokines IL-4, IL-5, and IL-13 after sensitization and 8-week-peanut food exclusion as well as during sustained oral exposure to peanuts compared with naïve mice (figures 3B, 3C, and 3D). The Th1 cytokine IFN-γ also significantly increased after sensitization during sustained oral exposure to peanuts (p<0.001 or p<0.01, figure 3E). It was verified that the cytokine profiles of the same cells cultured in the presence or absence of concanavalin A did not differ between groups (data not shown). No secretion was seen when cells from naive and sensitized mice were cultured in medium alone.

### Effects of sustained oral exposure to peanuts in sensitized mice treated with EPIT

**Figure 4:**
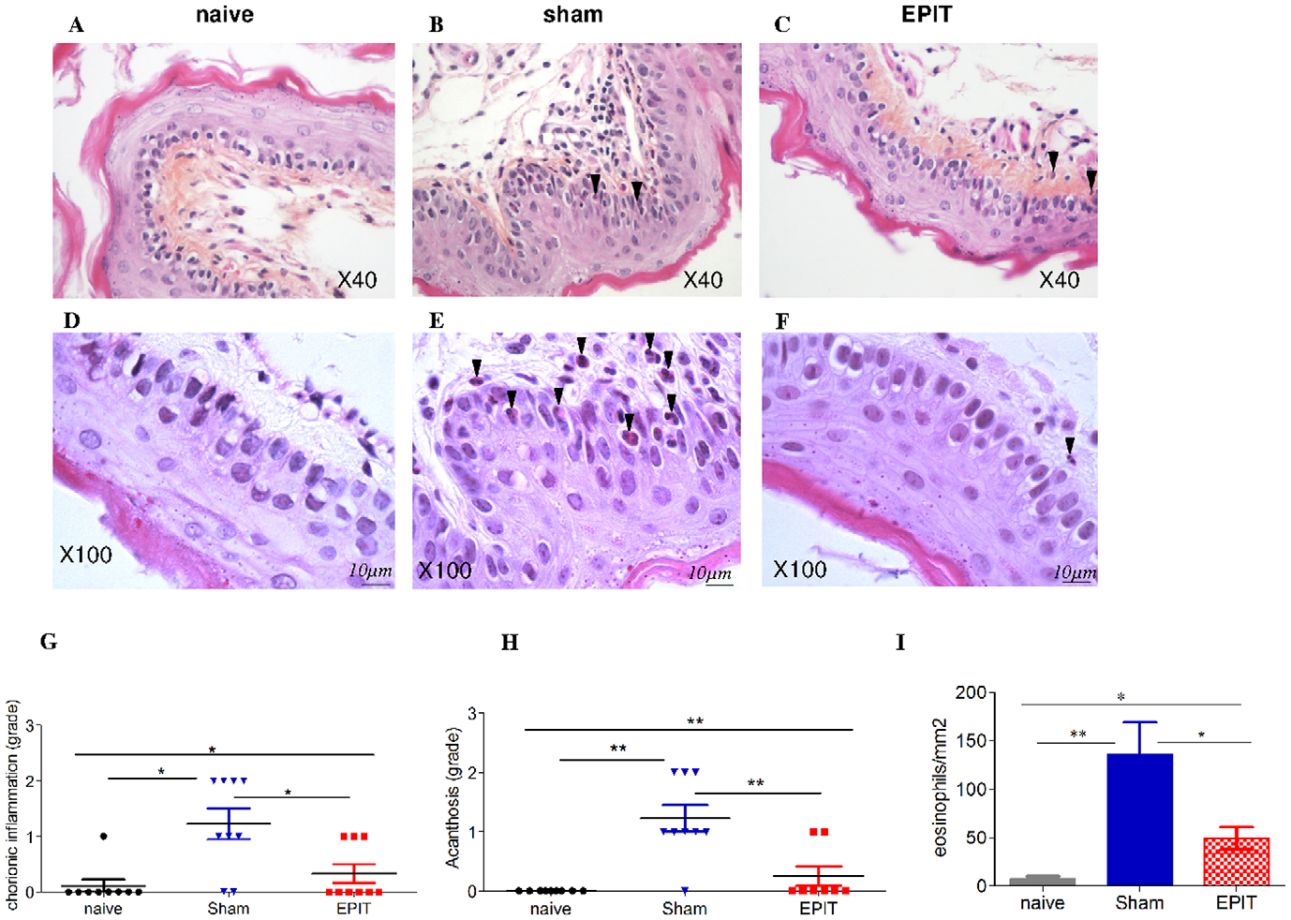
Effect of EPIT on the induction of peanut-induced allergic esophagus inflammation established by histological and RT-qPCR analyses Microscopic analysis of eosinophils in the esophagus at 40× (A–C) and 100× high-powered fields (D–F). Most eosinophils (arrows) are located in the lamina propia, submucosa, and epithelial layer of the sham group and to a lesser extent of the EPIT group. A difference in the thickness of epithelium is observed between naive/EPIT and sham. Analysis of (G) chorion inflammation, (H) acanthosis, (I) measurement of eosinophil infiltration in the esophagus and in 40× high-powered fields. Grading for chorion inflammation is as follows: 0 = nothing, 1 = slight diffuse infiltration, 2 = perivascular area. Grading for acanthosis is as follows: 0 = nothing, 1 = layer with more than 4 cells, 2 = layer with more than 6 cells. The epithelium thickness was expressed as mean (µm) ±SD. For eosinophils, the results are expressed as number of eosinophils per mm^2^ (means ± SD). Cytokine mRNA from esophagus segments collected 24 h after stopping peanut diet was assayed by RT-qPCR. Results are presented as mRNA expression of naive, Sham or EPIT animals. The relative levels of gene expression were calculated by reference to the mRNA levels of SDHA and β-actin in each sample. (J) eotaxin, (K) IL-5, (L) IL-13, (M) Foxp3, (N)GATA-3, (O) Tbet. * p<0.05, ** p<0.01.

In this model of esophago-gastro-enteropathy, EPIT was intercalated, 7 days after sensitization and 7 days before sustained oral exposure to peanut, allowing comparison of all groups (naive mice, sham, and EPIT treatments). In EPIT mice (Figure 4), the tissue sections following sustained peanut food exposure exhibited a lower cell infiltration in the lamina propria and epithelium than in Sham, with an aspect similar to naive mice. Semi quantitative evaluation of inflammation was lower in EPIT than Sham (0.33 vs 1.22, p<0.05; Figure 4G) as it was acanthosis (0.25 vs 1.22, p<0.01; Figure 4H). The thickness of the epithelium was also significantly decreased in EPIT (data not shown). Moreover, eosinophilic infiltration of the mucosa was clearly smaller in EPIT than in Sham, (49.6 *vs* 136.2 eosinophils/mm^2^, p<0.05 vs sham). At the molecular level, EPIT prevented induction of Th2 cytokines (Figures 4J, 4K, and 4L), as indicated by significantly lower mRNA levels *vs* Sham for eotaxin (p<0.05), IL-5 (p<0.05), and IL-13 (p<0.05). The mRNA levels following EPIT were similar to those in naive mice. The expression of *Foxp3* was significantly higher after EPIT compared with the Sham and naive groups (p<0.05) (figure 4M). Moreover, EPIT induced a significant decrease of the Th2 transcription factor GATA-3 compared with the Sham-treated group (p<0.05) (figure 4N) and had no effect on the Th1 transcription factor Tbet (Figure 4O). No significant changes were observed for mRNA levels of IL-4, IL-10 and IFN-γ (data not shown).

**Figure 5:**
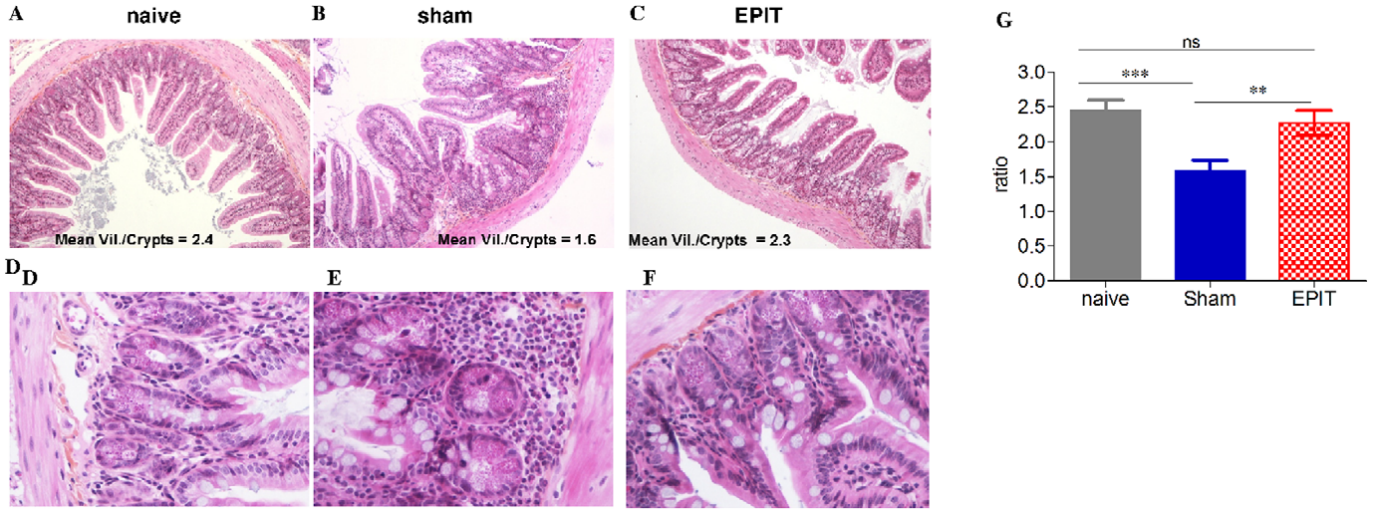
Effect of EPIT on the induction of peanut-induced allergic enteropathy. (A–F) Jejunum segments collected and analyzed by microscopy after HES coloration (×40). Upper layer: villous atrophia could be observed in the sham group. Lower layer: inflammatory infiltration, particularly of eosinophils is shown. (G) Measurement of the ratio of villous height by crypt depth for each group of 10 mice under 10× high-powered fields. Results are expressed as means ± SD. ns: non significant, ** p<0.01, *** p<0.001.

After EPIT, the tissue sections of the jejunum obtained following the peanut exclusive diet showed a sub-mucosal eosinophilic infiltration that was reduced compared with Sham (Figures 5A–5F). EPIT also prevented modification of the villous height/crypt depth ratio observed with sham (Figure 5G). Indeed, the decrease in height/crypt ratio was abolished (2.3±0.18, p<0.01 *vs* Sham), and the ratio was similar to that observed in naive mice.

**Figure 6:**
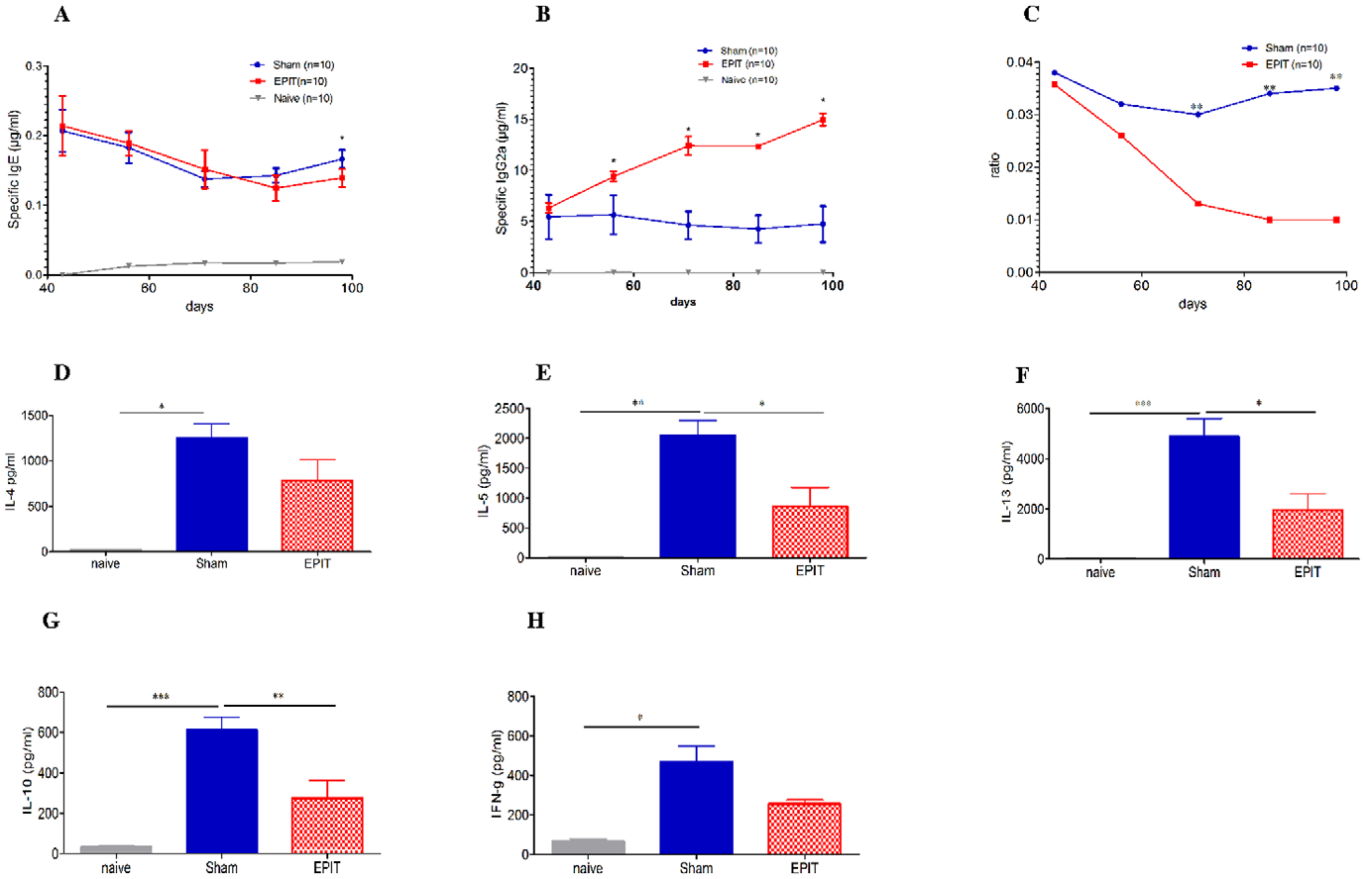
Effect of EPIT on systemic response induced in mice after oral sensitization. (A) Quantity of specific IgE expressed in µg.ml^−1^ for each group. (B) Quantity of specific IgG2a expressed in µg.ml^−1^ for each group. (C) Determination of the IgG1/IgG2a ratio expressed for each group. D44 (week 6) concords with the end of sensitization and from D44 to D99 (weeks 7 to 14) with the immunotherapy. Data are expressed as means ± SD for each group of 10 mice. (D–H) Measurement of Th2 cytokine levels (IL-4, IL-5, IL-10, IL-13) and IFN-γ secretion by splenocytes collected from each group of mice (EPIT, Sham, and naïve) immediately after sacrifice. Splenocytes were prepared and stimulated with PPE for 72 h. Cytokines were measured by ELISA. Data are presented as means ± SD for each group of 10 mice. ns: non significant, * p<0.05, ** p<0.01 and *** p<0.001.

Specific antibodies (sIgE, sIgG1, and sIgG2a) were monitored after sensitization and every 2 weeks during the 8 weeks of epicutaneous treatment (Figures 6A, 6B and 6C). During the 8 weeks of EPIT, sIgE slowly decreased until there was a significant difference between EPIT and Sham (p<0.05). Similarly, sIgG2a significantly increased in EPIT compared with Sham-treated mice (p<0.05) from the 2^nd^ week until the end of the immunotherapy (Figure 6B). No changes were observed in Sham-treated mice. Specific IgG1 were not modified after the 8 weeks of EPIT (data not shown). The sIgG1/sIgG2a ratio (Figure 6C) significantly increased during the 8 weeks of immunotherapy in EPIT-treated mice, with no modification in Sham-treated mice, as previously reported [8], [9]. Moreover, splenocytes from EPIT-treated mice produced significantly less IL-5 and IL-13 (p<0.05), and IL-10 (p<0.01) compared with the Sham group. The Th1 cytokine IFN-γ was not influenced by PPE treatment and the same applied to TGF-β secreted by Treg cells (data not shown).

## Discussion

This is the first study to assess the efficacy of EPIT on the esophago-gastro-enteropathy triggered by a sustained oral exposure to peanuts. The effects of food on the esophagus [15]–[19] and jejunum [20] in sensitized mice have been already evaluated in models of eosinophilic esophagitis published by Mishra *et al.* [15]–[17], using fungus or house dust mite sensitization followed by nasal or oral challenge. Mice sensitized then challenged to allergens exhibited severe mucosal injury with chorionic infiltration by inflammatory cells including eosinophils as well as acanthosis in the esophageal mucosa. These authors did not use peanut allergens neither orally exposed sensitized animals. In contrast, we maintained in a peanut-free diet previously sensitized animals in order to more closely mimic the elimination diet which is prescribed to EGID patients [21]. Along this line, in our model the allergen was subsequently reintroduced, to further reproduce these clinical recommendations [12]. Sustained oral exposures to peanuts allowed us to obtain the first model of combined esophageal and jejunal injuries. The esophagus epithelium was thickened, mainly due to hyperplasia of the acanthocytic layer. Mucosal RT-qPCR showed significantly higher mRNA levels of eotaxin, IL-5, and IL-13 which are involved in the pathogenesis of EoE [18], [22], [23]. In particular, IL-5 and eotaxin are involved in chemotaxis of eosinophils whereas IL-13 interacts with epithelial-differentiation cluster genes which determine tissue remodeling in human EoE [23], [24]. These results, in parallel with the measurement of cytokines in the supernatant of *in vitro* reactivated spleen cells after provocation by PPE, are consistent with the data summarized by Rothenberg *et al.* [25] and recently published by his coworkers [23], [24], [26], which confirmed the increase of these cytokines during human EoE. In our analysis of the kinetics of induction of digestive lesions by peanut regimen, histological data and IL-5 mRNA expression showed that digestive lesions were the most marked after 10 days of sustained oral peanut exposure. In the jejunum, sustained oral exposure to peanuts in sensitized mice induced a villus sub-atrophy combined with a high recruitment of eosinophils in the lamina propria. These digestive lesions closely resemble those described in EGID, [2]. They also resemble the lesions seen in food-induced malabsorption syndrome [27], which seems to become less frequent. The method allows an exact quantification of digestive lesions, thus facilitating the evaluation of any kind of treatment. Compared to the studies published by Mishra et al. [15]–[17], [19], our mice had higher levels of eosinophil infiltrations likely due to peanut diet re-challenge (Mishra et al. used saline) and the conventional housing used (versus SPF conditions).

Despite the remarkable severity of lesions induced by the peanut regimen, they were almost fully prevented by EPIT, which seem to render animals almost insensitive to sustained oral peanut exposure. These results agree with our previous reports on EPIT efficacy on airway hyper-responsiveness in mice sensitized to peanut proteins and treated with EPIT before aerosol challenge [9], invasive and non invasive measurements of airway hyper-responsiveness showed that EPIT-treated mice were comparable to naive mice, with values lower than in non-treated animals [9]. In the present study, all parameters linked to esophageal injuries, *i.e.* chorionic inflammation, acanthosis, and eosinophilic infiltration, and to villus sub-atrophy, were significantly reduced by EPIT to the levels observed in naive mice. Further confirmation is given by the decrease of mRNA expression for Th2 cytokines as IL-5, IL-13, and a chemoattractant of eosinophils, eotaxin, all involved in the pathogenesis of EoE. The decrease of inflammation in the esophagus might be a consequence of the downregulation of Th2 cytokines by regulatory mechanisms: the increased expression of Foxp3 mRNA in esophageal mucosa might downregulate the expression of GATA-3 mRNA, a Th2 transcription factor. On the other hand, EPIT had no effect on the expression of Tbet mRNA. Similar effects were obtained in the jejunum with a complete protection of the villus. A preliminary approach to identify the mechanism was made. In a first experiment of depletion of CD4^+^CD25^+^ regulatory T cells in sensitized mice during EPIT, the high infiltration of eosinophils after the sustained peanut oral exposure was maintained. Moreover, the protective effect of EPIT was transferrable by isolated regulatory T cells from spleen of mice [28].

Thus, we have shown here that EPIT using Viaskin® loaded with 100 µg of PPE and applied weekly over 48 h for 8 consecutive weeks before sustained oral exposure to peanuts in sensitized mice prevents the development of any digestive injuries. This contrasts with the observations of Akei *et al.* [29] whose studies suggest that epicutaneous exposure to allergens could prime for digestive injuries, especially EoE, *via* a Th2-dependant mechanism. Moreover, in the present study, peripheral sIgE and sIgG2a, monitored every two weeks, showed with EPIT a rapid increase of sIgG2a and a more gradual, slight decrease of sIgE. sIgG1 remained unchanged. A previous immunotherapy study in peanut-sensitized mice [30], showed a similar reduction of peanut-specific IgE levels throughout the treatment, accompanied by a persistent elevation in sIgG2a, a subclass of IgG which acts as a blocking antibody and interferes with mast cell degranulation *via* FcεRI ligation and antigen interception [31]. Thus, in addition to sustained reduction of peanut-specific IgE, increased peanut-specific IgG2a may contribute to the benefits of treatment with EPIT. At the cellular level, we also found that EPIT reduced the splenocyte secretion of IL-4, IL-5, IL-10, and IL-13 produced by CD4^+^ Th2-cells, which play a central role in the pathogenesis of allergic disorders [32]. In contrast, the Th1 cytokine IFN-γ was not modulated by EPIT, suggesting that the treatment established a more balanced Th1/Th2 ratio. Epicutaneous application of allergens onto intact skin of sensitized mice leads to its transport *via* dendritic cells to the draining lymph node. The migration of allergen-loaded professional APC regulated by a complex interplay of soluble and membrane-bound signals is likely to promote the specific regulatory T cell response not only at the draining lymph node but [33], but also at the systemic level, thus reaching the gut immune system [34].

In conclusion, we developed an original model of sustained oral exposure to peanuts resulting in EoE combined with villus sub-atrophy in sensitized animals. This model was then used to assess the efficacy of EPIT, which rendered peanut-sensitized mice almost insensitive to the peanut regimen. This study may open the way to a specific immunotherapy approach in allergy-induced diseases especially EoE and enteropathy. Further studies will be mandatory to confirm that EPIT is the most effective method.
